# Comparative assessment of native and heterologous 2-oxo acid decarboxylases for application in isobutanol production by *Saccharomyces cerevisiae*

**DOI:** 10.1186/s13068-015-0374-0

**Published:** 2015-12-01

**Authors:** N. Milne, A. J. A. van Maris, J. T. Pronk, J. M. Daran

**Affiliations:** Department of Biotechnology, Delft University of Technology, Julianalaan 67, 2628 BC Delft, The Netherlands

**Keywords:** *Saccharomyces cerevisiae*, 2-oxo acid decarboxylase, *Lactococcus lactis*, Isobutanol production, Fusel alcohol production

## Abstract

**Background:**

Decarboxylation of α-ketoisovalerate to isobutyraldehyde is a key reaction in metabolic engineering of *Saccharomyces cerevisiae* for isobutanol production with published studies relying on overexpression of either the native *ARO10* gene or of the *Lactococcus lactis kivD* decarboxylase gene resulting in low enzymatic activities. Here, we compare relevant properties for isobutanol production of Aro10, KivD and an additional, less studied, *L. lactis* decarboxylase KdcA.

**Results:**

To eliminate interference by native decarboxylases, each 2-oxo acid decarboxylase was overexpressed in a ‘decarboxylase-negative’ (*pdc1*Δ *pdc5*Δ *pdc6*Δ *aro10*Δ) *S. cerevisiae* background. Kinetic analyses in cell extracts revealed a superior *V*_max_/*K*_m_ ratio of KdcA for α-ketoisovalerate and a wide range of linear and branched-chain 2-oxo acids. However, KdcA also showed the highest activity with pyruvate which, in engineered strains, can contribute to formation of ethanol as a by-product. Removal of native decarboxylase genes eliminated growth on valine as sole nitrogen source and subsequent complementation of this growth impairment by expression of each decarboxylase indicated that based on the increased growth rate, the in vivo activity of KdcA with α-ketoisovalerate was higher than that of KivD and Aro10. Moreover, during oxygen-limited incubation in the presence of glucose, strains expressing *kdcA* or *kivD* showed a ca. twofold higher in vivo rate of conversion of α-ketoisovalerate into isobutanol than an *ARO10*-expressing strain. Finally, cell extracts from cultures grown on different nitrogen sources revealed increased activity of constitutively expressed KdcA after growth on both valine and phenylalanine, while KivD and Aro10 activity was only increased after growth on phenylalanine suggesting a difference in the regulation of these enzymes.

**Conclusions:**

This study illustrates important differences in substrate specificity, enzyme kinetics and functional expression between different decarboxylases in the context of isobutanol production and identifies KdcA as a promising alternative decarboxylase not only for isobutanol production but also for other branched-chain and linear alcohols.

**Electronic supplementary material:**

The online version of this article (doi:10.1186/s13068-015-0374-0) contains supplementary material, which is available to authorized users.

## Background

The yeast *Saccharomyces cerevisiae* is used for the industrial production of fuel ethanol, the largest single product in industrial biotechnology. In comparison with ethanol, isobutanol has chemical properties that make it a superior fuel for several engine types [1]. Moreover, isobutanol is an interesting precursor for a variety of products [2]. While *S. cerevisiae* naturally produces isobutanol from sugars [3, 4], titres in wild-type cultures are very low. Its high glycolytic flux, ability to grow anaerobically and robustness in industrial processes (including its insensitivity to phage infection), has stimulated an intensive research effort in industry and academia to engineer this yeast for high-yield isobutanol production [5–12].

In *S. cerevisiae*, isobutanol is a natural product of valine catabolism via the Ehrlich pathway [3, 4]. In this pathway, valine is first transaminated to yield 3-methyl-2-oxobutanoate (α-ketoisovalerate, KIV), which is subsequently decarboxylated to isobutyraldehyde, whose NAD(P)H-dependent reduction by a yeast alcohol dehydrogenase yields isobutanol. Engineering *S. cerevisiae* for fast, efficient and anaerobic conversion of sugars into KIV, a natural intermediate of valine biosynthesis, involves major challenges, for example related to redox-cofactor balancing, subcellular compartmentation of key enzymes and iron-sulphur-cluster assembly in the Ilv3 protein [6]. Perhaps because of the magnitude of these challenges, the subsequent step in isobutanol production, the decarboxylation of KIV, has received comparatively little attention in scientific literature.

*S. cerevisiae* contains four native thiamine-pyrophosphate-dependent 2-oxo acid decarboxylases, of which Pdc1, Pdc5 and Pdc6 encode pyruvate decarboxylase enzymes with a preference for linear-chain 2-oxo acids (pyruvate, 2-oxobutanoate and 2-oxopentanoate) [13]. While the three Pdc isoenzymes exhibit a low activity with KIV, their much higher activity and affinity for pyruvate [13] renders them unsuitable for high-efficiency production of isobutanol and other fusel alcohols. In contrast, Aro10 has been reported to have no activity with pyruvate and a much higher activity for the 2-oxo acid intermediates in fusel alcohol production [14]. For example, Aro10 decarboxylates phenylpyruvate that is formed during phenylalanine degradation and plays a key role in yeast-based production of phenylethanol, an important aroma compound [15]. However, *ARO10* is only transcribed during growth with aromatic, branched-chain or sulphur-containing amino acids as the nitrogen source [16, 17]. When ammonium sulphate is the nitrogen source, the wild-type *ARO10* gene is not transcribed and even the expression of *ARO10* from a constitutive promoter yields minimal enzyme activity indicating an as yet unknown mechanism of post-transcriptional regulation [13, 14].

Despite the low activity of Aro10 in cultures grown on simple nitrogen sources (such as urea and ammonium), constitutive overexpression of *ARO10*, combined with overexpression of valine biosynthesis genes has been used in metabolic engineering studies on isobutanol production by *S. cerevisiae* [6, 9]. Expression in *S. cerevisiae* of the *kivD* gene from *Lactococcus lactis* IFPL730, which encodes a 2-oxo acid decarboxylase, has also been used in several studies on isobutanol production [7, 8, 10, 11, 18] and yielded higher isobutanol titres than expression of Aro10 [10]. However, a quantitative comparison of these two decarboxylases is complicated by the simultaneous overexpression of other enzymes in the isobutanol pathway and by the presence of native 2-oxo acid decarboxylases (Pdc1, Pdc5, Pdc6, Aro10), and the use of complex media containing valine (a precursor for isobutanol production) and aromatic amino acids (which can induce *ARO10* activity).

KivD is not the only 2-oxo acid decarboxylase found in *L. lactis.* KdcA, identified in *L. lactis* B1157, has not yet been expressed in *S. cerevisiae* but has been used for engineering isobutanol production in *E. coli* [19]. The genes (*kivD* vs *kdcA*) and proteins (KivD vs KdcA) exhibit 85 and 87 % identity at the DNA and protein level, respectively, suggesting that this enzyme may be a promising alternative in the context of isobutanol production. While a preliminary characterization of the substrate specificities of KivD and KdcA has been performed previously by expression in bacterial hosts [18, 20], a quantitative analysis of their performance after expression in *S. cerevisiae* is not available.

In view of the industrial relevance of yeast-based isobutanol production and the essential role of decarboxylation in this process, the goal of the present study is to evaluate the suitability of the ‘novel’ 2-oxo acid decarboxylase KdcA from *L. lactis* B1157 [20], the frequently used KivD from *L. lactis* IFPL730 [18], and the native *S. cerevisiae* 2-oxo acid decarboxylase Aro10 [14] for metabolic engineering strategies aimed at constructing efficient isobutanol-producing *S. cerevisiae* strains. To this end, each 2-oxo acid decarboxylase was expressed from a strong constitutive promoter in a ‘decarboxylase-negative’ (*pdc1*Δ, *pdc5*Δ, *pdc6*Δ, *aro10*Δ) *S. cerevisiae* strain background. However, pyruvate decarboxylase-negative (*pdc1*Δ, *pdc5*Δ, *pdc6*Δ) strains (Pdc^−^) cannot grow on high glucose concentrations and also require C2-compounds (e.g. ethanol) for growth with low glucose concentrations. An evolutionary engineering strategy was used to identify a suppressor mutation to that phenotype [21]. Therefore, to circumvent glucose sensitivity, a mutant allele of *MTH1* that encodes a negative regulator of the glucose-sensing signal transduction pathway which contains an 225 bp internal deletion was introduced to restore growth of the resulting Pdc^−^ strains on glucose [21]. To evaluate and compare the three decarboxylases, an in vitro kinetic analysis was performed with a range of branched-chain and linear-chain 2-oxo acids. In vivo functionality was assessed by monitoring growth of ‘single-decarboxylase’ strains on several amino acids, whose catabolism proceeds via an Ehrlich pathway, as sole nitrogen sources. Finally, to test in vivo activity of the decarboxylases in isobutanol production, bioconversion of α-ketoisovalerate by the single-decarboxylase strains was studied in oxygen-limited cultures.

## Results

### In vitro enzymatic analysis of 2-oxo acid decarboxylase overexpression

The substrate specificity of the decarboxylases encoded by *kivD* and *kdcA* has been previously analysed using purified enzyme isolated from *L. lactis* IFPL730 (*kivD*) and using cell extracts via overexpression in *E. coli* (*kdcA*) [18, 20]. Despite their potential relevance for engineering of isobutanol-producing yeast, these enzymes have not previously been characterized upon expression in *S. cerevisiae*. We therefore compared their kinetic properties, not only for α-ketoisovalerate, but also for a wide range of branched-chain and linear-chain 2-oxo acids, with those of the native *S. cerevisiae* 2-oxo acid decarboxylase Aro10 in cell extracts of *S. cerevisiae* strains that expressed individual decarboxylase genes under the control of a strong, constitutive promoter.

The ‘single-decarboxylase’ *S. cerevisiae* strains IME260 (*pdc1*Δ *pdc5*Δ *pdc6*Δ *MTH1ΔTaro10*Δ *ARO10*↑), IME261 (*pdc1*Δ *pdc5*Δ *pdc6*Δ *MTH1ΔTaro10*Δ *kdcA*↑), IME262 (*pdc1*Δ *pdc5*Δ *pdc6*Δ *MTH1ΔTaro10*Δ *kivD*↑) and the decarboxylase-negative control strain IME259 (*pdc1*Δ *pdc5*Δ *pdc6*Δ *MTH1ΔTaro10*Δ p426GPD) were grown in 1-L shake flasks, containing 200 mL SME medium until mid-exponential phase (~OD 4.0), followed by the preparation of cell extracts for enzyme activity assays. Ethanol was chosen as a carbon source to minimize risks of evolution of the decarboxylases towards the use of pyruvate as a substrate. Decarboxylase activity was assayed with, phenylpyruvate, α-ketoisovalerate, α-ketomethylvalerate, α-ketoisocaproate, 4-methylthio-2-oxobutanoate, 2-oxobutanoate, 2-oxopentanoate and pyruvate, 2-oxo acids intermediates of phenylalanine, valine, isoleucine, leucine, methionine, threonine, norvaline and ethanol metabolism, respectively. Decarboxylase activity was not observed for any of the substrates tested with cell extracts of strain IME260 (Aro10) or IME259 (decarboxylase-negative strain). These results confirm the earlier observation that Aro10 is not active in media containing ammonium sulphate as sole nitrogen source and that no other 2-oxo acid decarboxylases operate in strain IME259 [13]. Kinetic parameters (*V*_max_ and *K*_m_) of KdcA (strain IME261) and KivD (strain IME262) were estimated by fitting kinetic data using non-linear regression of both the Michaelis–Menten and Hill equation. Clear Hill-type cooperativity was only observed for decarboxylation of KIV by KivD (Table 1). In all other cases, estimated Hill coefficients were below 1.25 and, consequently, *V*_max_ and *K*_m_ values were calculated by fitting experimental data to the Michaelis–Menten equation.

**Table 1:** Decarboxylation kinetics of branched-chain, aromatic, sulphur-containing and linear 2-oxo acids by cell extracts of *S. cerevisiae* strains expressing single 2-oxo acid decarboxylase genes

Substrate	Strain	*K*_m_ (mM)	*V*_max_ (U mg protein^−1^)	Hill coefficient (n)	*V*_max_/*K*_m_ (U mg protein^−1^ mM^−1^)
Pyruvate	IME259 (control)	BD	BD	NA	NA
	IME260 (*ARO10*↑)	BD	BD	NA	NA
	IME261 (*kdcA*↑)	33.0 ± 3.61	0.03 ± 0.00	1.2 ± 0.4	0.00091
	IME262 (*kivD*↑)	BD	BD	NA	NA
Phenylpyruvate	IME259 (control)	BD	BD	NA	NA
	IME260 (*ARO10*↑)	BD	BD	NA	NA
	IME261 (*kdcA*↑)	0.20 ± 0.04	0.12 ± 0.01	1.0 ± 0.1	0.60
	IME262 (*kivD*↑)	BD	BD	NA	NA
α-ketoisovalerate	IME259 (control)	BD	BD	NA	NA
	IME260 (*ARO10*↑)	BD	BD	NA	NA
	IME261 (*kdcA*↑)	8.31 ± 1.34	2.34 ± 0.25	0.9 ± 0.1	0.28
	IME262 (*kivD*↑)	7.73 ± 1.62^a^	0.03 ± 0.00^a^	2.7	0.0039
α-ketomethylvalerate	IME259 (control)	BD	BD	NA	NA
	IME260 (*ARO10*↑)	BD	BD	NA	NA
	IME261 (*kdcA*↑)	3.49 ± 0.34	0.69 ± 0.07	0.8 ± 0.1	0.20
	IME262 (*kivD*↑)	12.9 ± 2.87	0.05 ± 0.01	1.0	0.0039
α-ketoisocaproate	IME259 (control)	BD	BD	NA	NA
	IME260 (*ARO10*↑)	BD	BD	NA	NA
	IME261 (*kdcA*↑)	0.57 ± 0.09	0.49 ± 0.00	1.0	0.86
	IME262 (*kivD*↑)	2.42 ± 0.90	0.04 ± 0.01	1.2	0.017
4-methylthio-2-oxobutanoate	IME259 (control)	BD	BD	NA	NA
	IME260 (*ARO10*↑)	BD	BD	NA	NA
	IME261 (*kdcA*↑)	1.43 ± 0.22	0.13 ± 0.00	1.1	0.091
	IME262 (*kivD*↑)	BD	0.01 ± 0.00^b^	NA	NA
2-oxobutanoate	IME259 (control)	BD	BD	NA	NA
	IME260 (*ARO10*↑)	BD	BD	NA	NA
	IME261 (*kdcA*↑)	5.58 ± 0.58	0.10 ± 0.01	1.1	0.018
	IME262 (*kivD*↑)	BD	BD	NA	NA
2-oxopentanoate	IME259 (control)	BD	BD	NA	NA
	IME260 (*ARO10*↑)	BD	BD	NA	NA
	IME261 (*kdcA*↑)	1.44 ± 0.28	0.17 ± 0.00	1.1	0.12
	IME262 (*kivD*↑)	BD	BD	NA	NA

*V*_max_ and *K*_m_ values were estimated from non-linear fitting of data to the Michaelis–Menten equation or, where indicated, the Hill equation. The Hill coefficient (*n*) was calculated from the Hill equation, with *n* > 1 indicating positive cooperativity

*NA* not applicable, *BD* below detection limit of 0.008 U mg protein^−1^

^a^Calculated using the Hill equation

^b^Enzyme activity at 25 mM substrate concentration

Cell extracts containing KivD (strain IME262) did not display a detectable activity with pyruvate. In contrast, cell extracts containing KdcA (strain IME261) exhibited a low but significant pyruvate decarboxylase activity. KivD exhibited similar maximum activities towards α-ketoisovalerate (*V*_max_ = 0.03 ± 0.00 U mg protein^−1^), α-ketomethylvalerate (*V*_max_ = 0.05 ± 0.01 U mg protein^−1^), α-ketoisocaproate (*V*_max_ = 0.04 ± 0.01 U mg protein^−1^), as well as a lower activity towards 4-methylthio-2-oxobutanoate (*V*_max_ = 0.01 ± 0.00 U mg protein^−1^) (Table 1). Maximum activities of KdcA for these four substrates were an order of magnitude higher than those of KivD. *K*_m_ values of KivD and KdcA for α-ketoisovalerate were nearly identical, but KdcA showed a lower *K*_m_ towards the isoleucine and leucine-derived 2-oxo acids. While KdcA displayed the highest *V*_max_ with α-ketoisovalerate (2.34 ± 0.25 U mg protein^−1^), it also had a relatively high *K*_m_ (8.31 ± 1.34 mM) resulting in a lower overall affinity (*V*_max_/*K*_m_ = 0.28 U mg protein^−1^ mM^−1^). The highest affinity was observed for α-ketoisocaproate for both KdcA (*V*_max_/*K*_m_ = 0.86 U mg protein^−1^ mM^−1^) as well as KivD (*V*_max_/*K*_m_ = 0.017 U mg protein^−1^ mM^−1^). KdcA also displayed activity towards the linear-chain 2-oxo acids 2-oxopentanoate and 2-oxobutanoate (*V*_max_/*K*_m_ = 0.018 and 0.12 U mg protein^−1^ mM^−1^, respectively) while no activity with these substrates was found for KivD. These results indicate that, upon expression in yeast, KdcA has a much higher specific activity in cell extracts for α-ketoisovalerate, the key 2-oxo acid in isobutanol production, than KivD, as well as a broader substrate specificity.

### 2-oxo acid decarboxylase-dependent restoration of amino acid degradation

In *S. cerevisiae*, branched-chain and aromatic amino acid degradation is initiated by transamination. While the resulting 2-oxo acid cannot be assimilated, its irreversible decarboxylation generates a thermodynamic pull for transamination, while reduction or oxidation of the resulting aldehyde detoxifies it and facilitates its removal from the cell [3]. When these amino acids are used as the nitrogen source, due to the deletion of native 2-oxo acid decarboxylases, the resulting 2-oxo acids may accumulate inside the cell leading to potential toxic effects and a negative impact on the thermodynamic feasibility of the transamination reactions [3]. To test the ability of KdcA, KivD and Aro10 to function in vivo as the sole decarboxylase in amino acid degradation, aerobic specific growth rates in micro-titre plate of the decarboxylase-negative control strain IME259 (*pdc1*Δ *pdc5*Δ *pdc6*Δ *MTH1ΔTaro10*Δ, p426GPD), IME260 (*pdc1*Δ *pdc5*Δ *pdc6*Δ *MTH1ΔTaro10*Δ *ARO10*↑), IME261 (*pdc1*Δ *pdc5*Δ *pdc6*Δ *MTH1ΔTaro10*Δ *kdcA*↑) and IME262 (*pdc1*Δ *pdc5*Δ *pdc6*Δ *MTH1ΔTaro10*Δ *kivD*↑), as well as of decarboxylase-positive control IME140 (*PDC1PDC5PDC6ARO10* p426GPD) were measured in SMG medium containing 5 g/L of either valine, leucine, isoleucine, phenylalanine, methionine or (NH_4_)_2_SO_4_ as the sole nitrogen source.

**Fig. 1:**
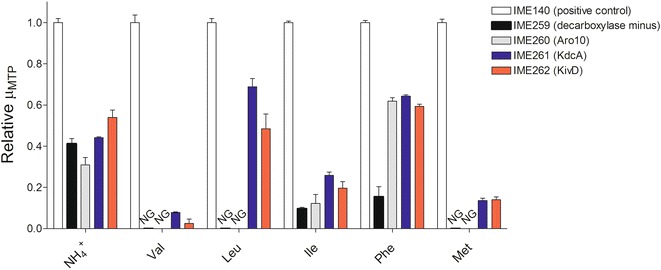
Restoration of amino acid catabolism in native ‘decarboxylase-negative’ background by 2-oxo acid decarboxylase expression. Relative specific growth rates in micro-titre plate (µ_MTP_) of IME140 (*PDC1 PDC5 PDC6 ARO10*) (*white bars*), IME259 (*pdc1*Δ, *pdc5*Δ, *pdc6*Δ, *aro10*Δ, *MTHΔT*, p426GPD) (*black bars*), IME260 (*pdc1*Δ, *pdc5*Δ, *pdc6*Δ, *aro10*Δ, *MTHΔT*, *ARO10*↑) (*grey bars*), IME261 (*pdc1*Δ, *pdc5*Δ, *pdc6*Δ, *aro10*Δ, *MTHΔT*, *kdcA*↑) (*blue bars*) and IME262 (*pdc1*Δ, *pdc5*Δ, *pdc6*Δ, *aro10*Δ, *MTHΔT*, *kivD*↑) (*red bars*) in SMG supplemented with 5 g/L (NH_4_)_2_SO_4_ (NH_4_^+^), valine (Val), leucine (Leu), isoleucine (Ile), phenylalanine (Phe) and methionine (Met). Cells were grown aerobically in 500 µL volumes in 48-well plates and incubated at 30 °C with OD 660 measured at 15 min intervals. Data are presented as averages and standard deviations of duplicate experiments relative to the average specific growth rate in micro-titre plate of IME140 in each nitrogen source. Specific growth rates in micro-titre plate for IME140 on each nitrogen source were as follows; NH_4_^+^: 0.33 ± 0.01 h^−1^, Val: 0.24 ± 0.01 h^−1^, Leu: 0.16 ± 0.00 h^−1^, Ile: 0.14 ± 0.00 h^−1^, Phe: 0.19 ± 0.00 h^−1^, Met: 0.18 ± 0.00 h^−1^. NG: No Growth

Consistent with the presence of a full complement of native decarboxylases, IME140 displayed the highest specific growth rate on all nitrogen sources tested. The inability of the decarboxylase-negative strain IME259 to grow on valine, leucine or methionine as the sole nitrogen source confirmed the crucial role of 2-oxo acid decarboxylation in the catabolism of these amino acids. IME259 grew slowly on isoleucine and phenylalanine, indicating that *S. cerevisiae* can tolerate the build-up of the corresponding 2-oxo acids and/or efficiently export them from the cells. The reduced growth rate of strain IME259 on ammonium-containing medium can be attributed to the role of *PDC1*, *PDC5* and *PDC6* in the fast conversion of glucose via alcoholic fermentation and indicates that the role of 2-oxo acid decarboxylases is not limited to amino acid catabolism. Growth rates of all other strains were therefore normalized to that of the decarboxylase-positive reference strain IME140 (Fig. 1).

Similar growth rates were obtained for the three single-decarboxylase strains when cultivated on ammonium sulphate. Expression of only Aro10 (strain IME260) stimulated growth only on phenylalanine (Fig. 1). This observation is consistent with a reported requirement for post-transcriptional regulation or activation by phenylalanine for its functional expression [16]. IME261 (*kdcA*↑) and IME262 (*kivD*↑) grew on all nitrogen sources tested. Although KdcA showed substantially higher *V*_max_ values than KivD towards all substrates tested, the difference in growth rate between strains overexpressing these enzymes was less prominent. A better correlation was found between growth rates on the different branched-chain amino acids and the in vitro *V*_max_/*K*_m_ ratios for the corresponding 2-oxo acids (Table 1). Growth studies and enzyme assays indicated that both *L. lactis* decarboxylases preferred α-ketoisocaproate (derived from leucine catabolism) over α-ketoisovalerate (derived from valine) and α-ketomethylvalerate (derived from isoleucine). While in vitro enzymatic activity of KivD for phenylpyruvate was below the detection limit of the assay, the comparatively high growth rate of IME262 on phenylalanine suggests that activity of KivD with this substrate may be higher in vivo.

### Nitrogen source-dependent 2-oxo acid decarboxylase activity

Expression of a gene from a constitutive promoter is not always sufficient to achieve high in vivo activity of the encoded protein. Earlier reports indicated that expression of Aro10 from a glycolytic promoter only yielded phenylpyruvate decarboxylase activity when *S. cerevisiae* was grown on phenylalanine [14, 16]. In the present study, phenylpyruvate decarboxylase activities in cell extracts of ammonium-grown cultures of the *kivD* overexpression strain IME262 were below detection limit, while its growth on phenylalanine was faster than on the other nitrogen sources tested. This observation prompted us to investigate whether phenylalanine-dependent decarboxylase activity also occurred for the *L. lactis* decarboxylases. Decarboxylation rates of pyruvate, phenylpyruvate and α-ketoisovalerate were measured in cell extracts of strains IME259 (control), IME260 (*ARO10*↑), IME261 (*kdcA*↑) and IME262 (*kivD*↑), grown on ammonium sulphate, phenylalanine or valine as sole nitrogen source (Table 2). Due to the absence of 2-oxo acid decarboxylases, IME259 was unable to grow in medium containing valine or phenylalanine. Strain IME260 did not grow in valine medium, indicating that valine cannot activate Aro10 in the same way as phenylalanine. Strains IME261(*kdcA*↑) and IME262 (*kivD*↑) grew on all nitrogen sources, indicating that they encoded a functional 2-oxo acid decarboxylase activity irrespective of the nitrogen source.

**Table 2:** 2-oxo acid decarboxylase activities in cell extracts of *S. cerevisiae* strains expressing single 2-oxo acid decarboxylases, grown on different nitrogen sources

Substrate	Strain	Nitrogen source		
		NH_4_^+^	Phenylalanine	Valine
Pyruvate	IME259 (control)	BD	NG	NG
	IME260 (*ARO10*↑)	BD	BD	NG
	IME261 (*kdcA*↑)	0.013 ± 0.001	0.036 ± 0.006	0.033 ± 0.005
	IME262 (*kivD*↑)	BD	0.012 ± 0.003	BD
Phenylpyruvate	IME259 (control)	BD	NG	NG
	IME260 (*ARO10*↑)	BD	0.046 ± 0.001	NG
	IME261 (*kdcA*↑)	0.045 ± 0.008	0.184 ± 0.029	0.155 ± 0.037
	IME262 (*kivD*↑)	BD	0.385 ± 0.040	0.013 ± 0.000
α-ketoisovalerate	IME259 (control)	BD	NG	NG
	IME260 (*ARO10*↑)	BD	0.056 ± 0.001	NG
	IME261 (*kdcA*↑)	0.683 ± 0.146	2.509 ± 0.509	1.900 ± 0.153
	IME262 (*kivD*↑)	0.093 ± 0.016	2.885 ± 0.107	0.151 ± 0.030

Enzyme activities, expressed as U mg protein^−1^, were determined at the following substrate concentrations: pyruvate: 50 mM, phenylpyruvate: 12.5 mM, α-ketoisovalerate: 25 mM

*NG* no growth, *BD* below detection limit of 0.008 U mg protein^−1^

No decarboxylase activity was detected in cell extracts of strain IME259 (negative control) and of IME260 (*ARO10*↑) grown on ammonium sulphate (Table 2). Cell extracts of strain IME260 grown on phenylalanine displayed decarboxylation activities with phenylpyruvate and α-ketoisovalerate, consistent with previous reports of phenylalanine-activated decarboxylase activity of Aro10 [13, 14]. Cell extracts of phenylalanine-grown cultures of the *kdcA* and *kivD* expressing strains IME261 and IME262 displayed a ca. threefold higher decarboxylase activity than ammonium sulphate grown cultures. In strain IME261 (*kdcA*↑), a similar high activity was found in cell extracts of valine-grown cultures. In contrast, cell extracts of valine-grown IME262 (*kivD*↑) showed no significant increase in activity relative to extracts of ammonium-grown cells.

### In vivo α-ketoisovalerate bioconversion by 2-oxo acid decarboxylases

To further investigate the in vivo activity of the three decarboxylases in a context more akin to an engineered, isobutanol-producing strain, we incubated micro-aerobic cell suspensions of different decarboxylase-expressing strains in the presence of α-ketoisovalerate (KIV) and glucose. After conversion of KIV to isobutyraldehyde by the tested 2-oxo acid decarboxylases, isobutanol formation in these experiments relies on the multiple yeast alcohol dehydrogenases that can reduce isobutyraldehyde [6]. Since many organic acids diffuse over the plasma membrane in their protonated form [22] and considering the pKa of KIV (3.37), experiments were performed at pH 3.5 and at 6.0 to test the effect of both protonated and deprotonated KIV. In these experiments, the decarboxylase-negative strain IME259 did not produce detectable levels of isobutanol or ethanol. Strain IME140, which harbours a full complement of native yeast 2-oxo acid decarboxylases produced both ethanol and isobutanol (Table 3). While pH had no significant impact on glucose consumption and ethanol production rates, KIV consumption and isobutanol production rates in all decarboxylase-expressing strains were at least twofold higher at pH 3.5 than at pH 6.0. In agreement with its high *V*_max_ in the in vitro enzyme activity measurements, IME261 (*kdcA*↑) exhibited the highest rate of isobutanol production, but also a higher ethanol production rate than strains IME260 (*ARO10*↑) and IME262 (*kivD*↑). This result is consistent with the pyruvate decarboxylase activity measured in vitro with KdcA (Table 1). While in vitro enzymatic activities of Aro10 and KivD were below the detection limit, very low rates of ethanol formation observed for both strains in the KIV bioconversion experiments suggest that these enzymes may have a low activity with pyruvate. Based on the ethanol production rates, enzyme activities of 0.71 ± 0.3 mU mg protein^−1^ and 3.7 ± 0.1 mU mg protein^−1^ were estimated for IME260 (*ARO10*↑) and IME262 (*kivD*↑), respectively (data for pH 3.5, calculations based on cellular protein content of 42 % [23]). These activities are below the detection limit of the enzyme assays (8 mU mg protein^−1^) while the estimated activity of KdcA was comparable to the in vitro value reported (8.1 ± 1.3 mU mg protein^−1^) (Table 4). Estimated in vivo enzyme activities of KIV decarboxylation were much lower than the activities measured in vitro. This result suggests that, in all strains, isobutanol production might have been limited by KIV uptake.

**Table 3:** α-ketoisovalerate bioconversion under micro-aerobic conditions by *S. cerevisiae* strains expressing different 2-oxo acid decarboxylase genes

Strain	Decarboxylase	Biomass-specific production/consumption rates (mmol/g biomass/h)							
		Glucose		Ethanol		KIV		Isobutanol	
		pH 6.0	pH 3.5	pH 6.0	pH 3.5	pH 6.0	pH 3.5	pH 6.0	pH 3.5
IME259	None	0.13 ± 0.01	0.15 ± 0.01	BD	BD	0.01 ± 0.00	0.04 ± 0.00	BD	BD
IME260	*ARO10*↑	0.16 ± 0.04	0.19 ± 0.00	0.02 ± 0.01	0.02 ± 0.01	0.04 ± 0.02	0.10 ± 0.00	0.04 ± 0.00	0.05 ± 0.00
IME261	*kdcA*↑	0.35 ± 0.03	0.35 ± 0.00	0.24 ± 0.00	0.20 ± 0.03	0.07 ± 0.00	0.17 ± 0.00	0.05 ± 0.00	0.10 ± 0.00
IME262	*kivD*↑	0.22 ± 0.01	0.22 ± 0.01	0.10 ± 0.01	0.10 ± 0.00	0.06 ± 0.01	0.12 ± 0.00	0.03 ± 0.00	0.07 ± 0.01
IME140	Wild-type control	2.04 ± 0.25	1.71 ± 0.04	2.50 ± 0.05	2.86 ± 0.08	0.08 ± 0.01	0.26 ± 0.03	0.03 ± 0.01	0.10 ± 0.00

Biomass-specific conversion rates were measured after addition of 10 g/L glucose and 100 mM α-ketoisovalerate (KIV) to cell suspensions at pH 6.0 and pH 3.5. Cells were incubated micro-aerobically (see “Methods”) and incubated at 30 °C. Data are presented as averages and mean deviations of duplicate experiments

*BD* below detection limit of HPLC

**Table 4:** Estimated in vivo activities of 2-oxo acid decarboxylases during α-ketoisovalerate (KIV) bioconversion experiments

Strain	Decarboxylase	Substrate	
		Pyruvate	KIV
IME259	None	BD	BD
IME260	*ARO10*↑	0.71 ± 0.29	2.02 ± 0.02
IME261	*kdcA*↑	8.07 ± 1.29	4.05 ± 0.05
IME262	*kivD*↑	3.74 ± 0.07	2.81 ± 0.38
IME140	Wild-type control	114 ± 3	3.83 ± 0.14

Pyruvate and KIV decarboxylase activities were estimated from ethanol and isobutanol production rates at pH 3.5, based on a biomass protein content of 42 % [23]. Activity is expressed in mU mg protein^−1^

*BD* below detection limit of HPLC

## Discussion

In metabolic engineering, knowledge on the kinetic properties and substrate specificity of (heterologous) enzymes used in product pathways is essential. For efficient isobutanol production by *S. cerevisiae* the 2-oxo acid decarboxylase should ideally combine: (1) high selectivity towards α-ketoisovalerate, which reduces competition for the active site and the formation of by-products (2) a high *V*_max_/*K*_m_ ratio, which enables fast conversion at low intracellular substrate concentrations, and in particular, (3) zero or very low activity with pyruvate, to prevent formation of ethanol as a major by-product. In this study, three 2-oxo acid decarboxylases that have previously been used in metabolic engineering of microbes for isobutanol production [13, 18, 20] were evaluated based on these criteria. Our data on in vitro enzyme kinetics and substrate specificity are in good agreement with a recent characterization of *S. cerevisiae* Aro10 [13]. They extend reports on the substrate specificity on KdcA and KivD expressed in the bacterial hosts *E. coli* and *L. lactis*, respectively, the data provided in this study showed similar substrate specificity trend for both KdcA and KivD [18, 20]. However, one notable deviation was the higher *K*_m_ of KivD for KIV observed in our results compared to previous reports (7.73 ± 1.62 mM vs. 1.9 mM). The difference in affinity might represent the difference in production host (*S. cerevisiae* vs *E. coli*) but also the sequence discrepancy since the purified enzyme from *E. coli* was also tagged with a N-terminus His_6_ tag.

The results show that while none of the three tested enzymes ideally met all three criteria, KdcA outperformed all other tested enzymes. KdcA displayed superior α-ketoisovalerate decarboxylase activity in in vitro assays (Table 1), as well as in the in vivo KIV bioconversion experiments (Table 3), but it also displayed the highest activity towards pyruvate, as illustrated by the in vivo KIV bioconversion experiments, this led to significant rates of ethanol production by a *kdcA* expressing strain. KivD displayed a lower activity towards pyruvate, but also supported lower rates of isobutanol production. Finally, Aro10 combined a very low activity for pyruvate with sub-optimal rates of KIV conversion and an, as yet unresolved, dependency on aromatic amino acids for full activity.

Our data indicate that, within this limited set of three decarboxylases, choice of an enzyme for isobutanol production in *S. cerevisiae* inevitably involves a compromise between KIV decarboxylation kinetics and formation of ethanol as a by-product. However, in view of the high *K*_m_ of KdcA for ethanol (33 ± 4 mM, Table 1) it may also be possible to reduce ethanol production by preventing the occurrence of high intracellular pyruvate concentrations. The first step in the isobutanol product pathway is the conversion of pyruvate to acetolactate by acetolactate synthase (mitochondrial Ilv2 in wild-type *S. cerevisiae*). When the entire isobutanol pathway is expressed in the cytosol [6], high-level expression of Ilv2 (*K*_m_ ca. 4 mM; [24]) or of bacterial acetolactate synthases with lower *K*_m_ values [25, 26] may keep cytosolic pyruvate concentrations sufficiently low to curtail pyruvate decarboxylation via KdcA.

Hill cooperativity has been reported for Pdc1, Pdc5 and Pdc6 [13, 27, 28] but not for Aro10 [14]. In this study, Hill cooperativity was only observed for KivD and with KIV as the substrate. Whether this requirement for substrate binding in order to activate the enzyme represents a drawback of KivD depends on intracellular concentrations of KivD in engineered strains.

Our results show that, as previously reported for Aro10 [14], expression from a constitutive promoter was not sufficient to achieve the highest activities of KivD and KdcA in media that contained ammonium sulphate as sole nitrogen source. KdcA activity was stimulated by growth with either valine or phenylalanine as the nitrogen source, while full activity of Aro10 and KivD activity was observed during growth on phenylalanine but not during growth on valine. The molecular mechanism for this post-transcriptional, nitrogen-source-dependent regulation has not yet been resolved. Understanding and, if possible, eliminating this level of regulation is a relevant goal in enabling robust, context-independent performance of decarboxylases in industrial isobutanol-producing strains.

While this study focused on the characterisation of 2-oxo acid decarboxylases potentially relevant for isobutanol production, the in vitro assays indicate that these enzymes may be applied in a wide range of alcohol production processes. In particular, KdcA displayed superior kinetic properties for a range of 2-oxo acids. Published studies on phenylethanol production [15] and 1-butanol production [12] use Aro10 and Pdc1, 5 and 6, respectively, to catalyse the decarboxylation of the relevant 2-oxo acid (phenylpyruvate and 2-oxopentanoate, respectively). However, this led either to a low activity (Aro10) or high rates of ethanol formation (Pdc1, 5 and 6). A comparison of data from the present study with a recent evaluation of the kinetics and substrate specificity of native *S. cerevisiae* decarboxylases [13] reveals KdcA performs at least as well as the native *S. cerevisiae* 2-oxo acid decarboxylases for all non-pyruvate substrates tested. In particular, KdcA significantly outperforms all Pdc isoforms with 2-oxopentanoate as substrate (132-fold higher *V*_max_/*K*_m_ than for pyruvate, as compared to 1.2-fold for the best performing native decarboxylase Pdc5; [13]). These data indicate that KdcA is an interesting enzyme for strategies to produce 1-butanol via the glyoxylate pathway [12].

Thiamine-pyrophosphate-dependent decarboxylases are widespread in nature suggesting that while the present study failed to find an ideal candidate enzyme for isobutanol production, scanning biodiversity for novel enzymes may be useful to identify better performing candidates, in particular, identifying variants with lower specificity towards pyruvate. A BLAST search using Aro10, KdcA and KivD protein sequences yielded over 90 sequences with sequence identity above 35 % including enzymes derived from eukaryotic micro-organisms not known to produce isobutanol and more importantly ethanol [29, 30]. A future strategy to improve KIV decarboxylation in *S. cerevisiae* might therefore involve high-throughput screening of a diverse range of heterologous decarboxylases using the methods described in this study. For example, by measuring the degree of complementation of heterologous 2-oxo acid decarboxylases in the presence of both glucose (measuring pyruvate affinity) and valine (measuring KIV affinity), a large number of novel candidate enzymes could be rapidly evaluated. A further potential strategy might involve protein engineering (e.g. gene shuffling) approaches [48]. In particular, future engineering should focus on optimizing *V*_max_ and *K*_m_ towards KIV, without a need for activation by specific amino acids and reducing the unwanted affinity towards pyruvate and other 2-oxo acids.

## Conclusions

Analysis of three 2-oxo acid decarboxylases for isobutanol production in *S. cerevisiae* revealed that based on in vitro enzymatic data, and in vivo complementation and α-ketoisovalerate bioconversion, while no one enzyme ideally meets our criteria for an optimum 2-oxo acid decarboxylase, KdcA outperformed all other enzymes tested and should be investigated further for application in isobutanol and other higher alcohol production strategies.

## Methods

### Media, strains and maintenance

All *S. cerevisiae* strains used in this study (Table 5) were derived from the CEN.PK genetic background [31, 32]. Frozen stocks of *E. coli* and *S. cerevisiae* were prepared by addition of glycerol [30 % (v/v)] to exponentially growing cells and aseptically storing 1 mL aliquots at −80 °C. Cultures were grown in chemically defined medium containing either ammonium sulphate or various amino acids as sole nitrogen source. Ammonium sulphate medium contained 3 g/L KH_2_PO_4_, 0.5 g/L MgSO_4_∙7H_2_O and 5 g/L (NH_4_)_2_SO_4_ [33]. Amino acid medium contained 6.6 g/L K_2_SO_4_, 3 g/L KH_2_PO_4_, 0.5 g/L MgSO_4_∙7 H_2_O and 5 g/L of the required amino acid. After supplementation of media with trace elements, vitamins and appropriate growth factors as described previously [34], the pH was adjusted to 6.0. Chemically defined medium with glucose as sole carbon source (SMG) contained 20 g/L glucose and chemically defined medium with ethanol as sole carbon source (SME) contained 20 mL/L ethanol. If required, 150 mg/L uracil was added to the media in order to complement a uracil auxotrophy [34]. For anaerobic growth Tween-80 (420 mg/L) and ergosterol (10 mg/L) were added. Defined medium plates were made with 20 g/L agar (Becton–Dickinson B.V. Breda, The Netherlands).

**Table 5:** *Saccharomyces cerevisiae* strains used in this study

Strain	Relevant genotype	Origin
IME140	MATa *ura3*-*52 HIS3 LEU2 TRP1 MAL2*-*8c SUC2* + p426GPD (2 µm *URA3*)	[31, 49]
CEN.PK707-4A	MATa *ura3*-*52 HIS3 LEU2 TRP1 MAL2*-*8c SUC2 pdc1::loxP pdc5::loxP pdc6::loxP*	[13]
IMI244	MATa *ura3*-*52 HIS3 LEU2 TRP1 MAL2*-*8c SUC2 pdc1::loxP pdc5::loxP pdc6::loxP MTH1∆T*	This study
IMI271	MATa *ura3*-*52 HIS3 LEU2 TRP1 MAL2*-*8c SUC2 ade2::PDC1_amdS*	This study
IMI275	MATa *ura3*-*52 HIS3 LEU2 TRP1 MAL2*-*8c SUC2 pdc1::loxP pdc5::loxP pdc6::loxP MTH1∆T ade2::PDC1_amdS*	This study
IMI302	MATa *ura3*-*52 HIS3 LEU2 TRP1 MAL2*-*8c SUC2 pdc1::loxP pdc5::loxP pdc6::cas*9-tagA-loxP-natNT2-loxP *MTH1∆T ade2::PDC1_amdS*	This study
IMK647	MATa *ura3*-*52 HIS3 LEU2 TRP1 MAL2*-*8c SUC2 pdc1::loxP pdc5::loxP pdc6::cas9*-tagA-loxP-natNT2-loxP *MTH1∆T aro10Δ*	This study
IME259	MATa *ura3*-*52 HIS3 LEU2 TRP1 MAL2*-*8c SUC2 pdc1::loxP pdc5::loxP pdc6::cas9*-tagA-loxP-natNT2-loxP *MTH1∆T aro10Δ* p426GPD	This study
IME260	MATa *ura3*-*52 HIS3 LEU2 TRP1 MAL2*-*8c SUC2 pdc1::loxP pdc5::loxP pdc6::cas9*-tagA-loxP-natNT2-loxP *MTH1∆T aro10Δ* pUDE001	This study
IME261	MATa *ura3*-*52 HIS3 LEU2 TRP1 MAL2*-*8c SUC2 pdc1::loxP pdc5::loxP pdc6::cas9*-tagA-loxP-natNT2-loxP *MTH1∆T aro10Δ* pUDE321	This study
IME262	MATa *ura3*-*52 HIS3 LEU2 TRP1 MAL2*-*8c SUC2 pdc1::loxP pdc5::loxP pdc6::cas9*-tagA-loxP-natNT2-loxP *MTH1∆T aro10Δ* pUDE336	This study
CEN.PK113-7D	MATa *URA3 HIS3 LEU2 TRP1 MAL2*-*8c SUC2*	[31, 32]
CEN.PK113-5D	MATa *ura3*-*52 HIS3 LEU2 TRP1 MAL2*-*8c SUC2*	[31, 32]
TAM	MATa *ura3*-*52 HIS3 LEU2 TRP1 MAL2*-*8c SUC2 pdc1Δ*::*loxP pdc5Δ*::*loxP pdc6Δ*::*loxP*, selected for C2 independence in glucose-limited chemostat cultures and glucose-tolerant growth in batch culture	[39]

### Strain and plasmid construction

PCR amplification was performed using Phusion^®^ Hot Start II High Fidelity Polymerase (Thermo scientific, Waltham, MA) according to manufacturer’s instructions using HPLC or PAGE purified, custom synthesized oligonucleotide primers (Sigma Aldrich, Zwijndrecht, The Netherlands) in a Biometra TGradient Thermocycler (Biometra, Göttingen, Germany). *L. lactis* B1157 *kdcA* [AY548760.1] and *L. lactis* IFPL730 *kivD* [AJ746364.1] open reading frames were codon optimized (co) for *S. cerevisiae* using the JCat algorithm [35], synthesized and cloned into pMA vectors (Amp^R^) resulting in pUD342 and pUD350, respectively (GeneArt, Bleiswijk, The Netherlands; Table 6, Additional file 1). To construct the overexpression plasmids pUDE321 (*TDH3*_*P*_-co*kdcA*-*CYC1*_*t*_) and pUDE336 (*TDH3*_*P*_-co*kivD*-*CYC1*_*t*_), co-*kdcA* and co-*kivD* were PCR amplified from pUD342 and pUD350, respectively, with primer pairs “KdcA fwd GPD_P_ homology/KdcA rev CYC1_T_ homology” and “KivD fwd GPD_P_ homology/KivD rev CYC1_T_ homology” (Table 7). The primers included a 5′ extension homologous to either the *TDH3* promoter or *CYC1* terminator regions of p426GPD [36] to allow for Gibson assembly with the vector backbone [37]. The p426GPD expression vector was digested with the restriction endonucleases SpeI and XhoI (Life Technologies Europe BV, Bleiswijk, The Netherlands), creating a linear vector backbone flanked by the *TDH3* promoter and *CYC1* terminator.

**Table 6:** Plasmids used in this study

Name	Characteristics	Origin
pUD342	*AmpR*, *E. coli* replicon, *COkdcA*	This study
pUD350	*AmpR*, *E. coli* replicon, *COkivD*	This study
p426GPD	2 µm ori, *URA3*, *TDH3*_*p*_-*CYC1*_*t*_	[36]
pUDE001	2 µm ori, *URA3*, *TDH3p*-*ARO10*-*CYC1t*	[14]
pUDE321	2 µm ori, *URA3*, *TDH3p*-*kdcA*-*CYC1t*	This study
pUDE336	2 µm ori, *URA3*, *TDH3p*-*kivD*-*CYC1t*	This study
pUG-AmdS	2 µm ori, *URA3*, *TEF2p*-*amdS*-*TEF2t*	[40]
pUG-natNT2	2 µm ori, *URA3*, *TEF2p*-*natNT2*-*TEF2t*	[45]
p414-pTEF1-Cas9-tCYC1	2 µm ori, *URA3*, *TEF1p*-*cas9*-*CYC1t*	[44]
pROS10	2 μm *URA3 gRNA*-*CAN1.Y gRNA*-*ADE2.Y*	[43]

*CO* codon optimized

**Table 7:** Oligonucleotide primers used in this study

Name	Sequence (5′ → 3′)
Primers for CRISPR-Cas plasmid assembly	
Plasmid backbone amplification	GATCATTTATCTTTCACTGCGGAGAAG
*ARO10* gRNA CRISPR KO sequence	TGCGCATGTTTCGGCGTTCGAAACTTCTCCGCAGTGAAAGATAAATGATCATTTACAAGTATTCTAAACCGTTTTAGAGCTAGAAATAGCAAGTTA AAATAAGGCTAGTCCGTTATCAAC
*ARO10* repair fragment upper	ACAAGTTGACGCGACTTCTGTAAAGTTTATTTACAAGATAACAAAGAAACTCCCTTAAGCAAACTTGTGGGCGCAATTATAAAACACTGCTACCAA TTGTTCGTTTTCTGTTCATTAACA
*ARO10* repair fragment lower	TGTTAATGAACAGAAAACGAACAATTGGTAGCAGTGTTTTATAATTGCGCCCACAAGTTTGCTTAAGGGAGTTTCTTTGTTATCTTGTAAATAAACT TTACAGAAGTCGCGTCAACTTGT
Primers for verification of knockouts	
PDC1 upstream fwd	AGCTGTCCTCGTTGAACATAG
PDC1 downstream rev	TTGCGTGAGGTTATGAGTAG
PDC5 upstream fwd	CAGAACCACCTACACTACC
PDC5 downstream rev	CTGGGTTCTTAGCATCCTTG
PDC6 upstream fwd	AACTCCCGCAAACAAAGGTG
PDC6 downstream rev	CAACACCTGCGAGATACCGTAG
Aro10 upstream Fwd	TGCTTGTACACCTCATGTAG
Aro10 downstream Rev	GCAGACATTTAGCAGATGTAG
Primers for verification of plasmid assembly and transformation	
GPD1 promoter Fwd	GGGATGTGCTGCAAGGCGATTAAGTTGG
CYC1 terminator Rev	GGCAGTGAGCGCAACGCAATTAATGTGAG
Primers for verification of genome integrations	
MTH1ΔT conformation fwd	CACCATGTTTGTTTCACCACCACCAGCAACTTCG
MTH1ΔT conformation rev	TCAGGATACTGAATCCGGCTGCCAATCCA
PDC1-AmdS at ADE2 conformation fwd	ATGTTATGCGCCTGCTAGAG
PDC1-AmdS at ADE2 conformation rev	ACATTCCGCCATACTGGAGG
Cas9-tag-natNT2 at PDC6 conformation fwd	AACTCCCGCAAACAAAGGTG
Cas9-tag-natNT2 at PDC6 conformation rev	CAACACCTGCGAGATACCGTAG
Primers for plasmid construction	
KdcA fwd GPD_P_ homology	CTACTTTTATAGTTAGTCTTTTTTTTAGTTTTAAAACACCAGAACTTAGTTTCGACGGATATGGATACAGTAGGAGATTACCTGTTAGACCG
KdcA rev CYC1_T_ homology	CGGTTAGAGCGGATGTGGGGGGAGGGCGTGAATGTAAGCGTGACATAACTAATTACATGACTATTTATTTTGCTCAGCAAATAATTTACCC
KivD fwd GPD_P_ homology	CTACTTTTATAGTTAGTCTTTTTTTTAGTTTTAAAACACCAGAACTTAGTTTCGACGGATATGTACACTGTTGGTGACTAC
KivD rev CYC1_T_ homology	CGGTTAGAGCGGATGTGGGGGGAGGGCGTGAATGTAAGCGTGACATAACTAATTACATGATTAAGACTTGTTTTGTTCAGCGAACAACTTACCC
Primers for cassette construction	
MTH1ΔT fwd	CACCATGTTTGTTTCACCACCACCAGCAACTTCG
MTH1ΔT rev	TCAGGATACTGAATCCGGCTGCCAATCCA
PDC1-ADE2 homology fwd	TTGCCCCAAGGCCTCACAACTCTGGACATTATACCATTGATGCTTGCGTCACTTCTCAATTTGAAGCTCATTTGAGATCAATATTGGATTTGCCAA TGCCTGCGACTGGGTGAGCATATG
PDC1-TEF2_T_ homology rev	CAGAAAGTAATATCATGCGTCAATCGTATGTGAATGCTGGTCGCTATACTGATCGGTTTTTTTTTTGGAAGACATCTTTTCC
AmdS cassette fwd	CAGTATAGCGACCAGCATTC
AmdS-ADE2 homology rev	GGCATTGGCAAATCCAATATTGATCTCAAATGAGCTTCAAATTGAGAAGTGACGCAAGCATCAATGGTATAATGTCCAGAGTTGTGAGGCCTTG GGGCAAGACATGGAGGCCCAGAATAC
PDC1-AmdS-ADE2 homology fwd	TCTAAGTACATCCTACTATAACAATC
PDC1-AmdS-ADE2 homology rev	CATTTGATGTAATCATAACAAAGCC
Cas9-PDC6 homology fwd	GTGCCTATTGATGATCTGGCGGAATGTCTGCCGTGCCATAGCCATGCCTTCACATATAGTCCGCAAATTAAAGCCTTCGAG
Cas9-tag homology rev	GCAGTCCTCTTTTATATACAGTATAAATAAAAAACCAGTAATATAGCAAAAACATATTGCCAGGGAACAAAAGCTGGAGCTCATAG
natNT2-tag homology fwd	GTGCCTATTGATGATCTGGCGGAATGTCTGCCGTGCCATAGCCATGCCTTCACATATAGTCCGCAAATTAAAGCCTTCGAG
natNT2-PDC6 homology rev	CAAACTGTGTAAATTTATTTATTTGCAACAATAATTCGTTTTTGAGTACACTACTAATGGCATAGGCCACTAGTGGATCTG

The expression vectors were assembled using Gibson assembly^®^ Master Mix (NEB, Ipswich, MA, USA) according to the manufacturer’s instructions. The assembly mix was then transformed into chemically competent *E. coli* (T3001, Zymo research, Irvine, CA, USA) according to the manufacturer’s instructions, and the gene sequences were confirmed by Sanger sequencing (BaseClear, Leiden, The Netherlands).

**Fig. 2:**
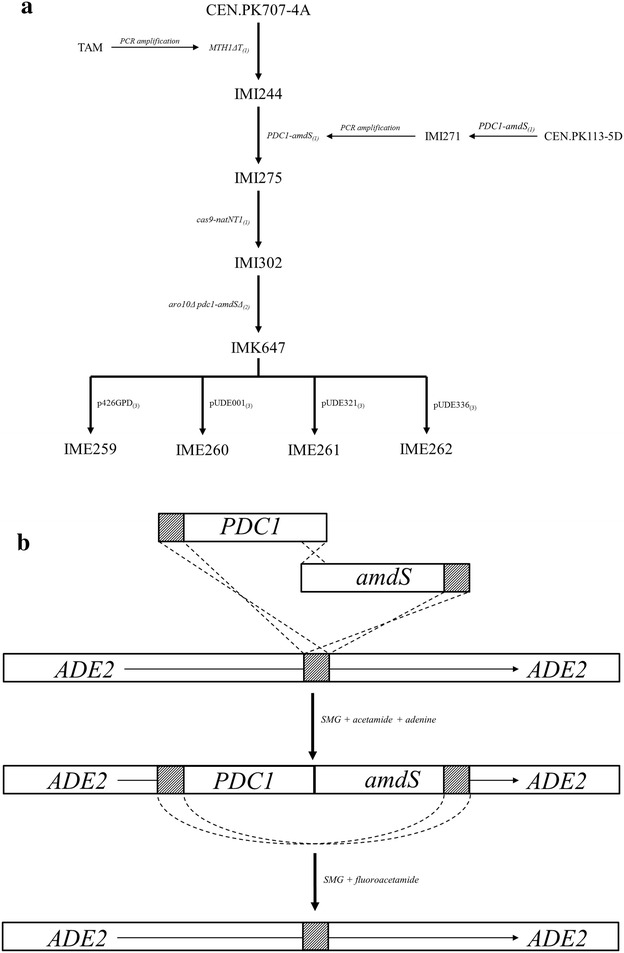
Overview of strain construction genealogy used in this study and transient *PDC1*-*amdS* expression cassette for targeted integration and rapid excision at the *ADE2* locus. **a** The order in which strains were constructed, as well as the modifications made either by (1) targeted integration of PCR product, (2) removal of gene or transient gene cassette or (3) introduction of a gene encoding plasmid. **b** The transient *PDC1* and *amdS* expression cassettes containing homology to the other respective cassette and to the *ADE2* locus were transformed into IMI244 allowing homologous assembly into a full-length cassette and targeted integration at the *ADE2* locus when plated on selective media containing acetamide (0.6 g/L) and adenine (20 mg/L) (resulting in IMI275). The resulting integration cassette was flanked by identical tags which have homology to the *ADE2* locus such that when plated on selective media containing fluoroacetamide (2.3 g/L) and the absence of adenine, removal of the cassette was induced resulting in reassembly of a functional *ADE2* gene (resulting in IMK647)

The 2-oxo acid decarboxylase-negative background strain IMK647 (*pdc1*Δ, *pdc5*Δ, *pdc6*Δ, *aro10*Δ *MTH1ΔT*) was constructed by first introducing the *MTH1ΔT* allele which restores growth on glucose [21] into CEN.PK707-4A (*pdc1*Δ, *pdc5*Δ, *pdc6*Δ) [13] using the pop in/pop out method [38]. This resulted in strain IMI244. The *MTH1ΔT* integration fragment was generated by PCR using genomic DNA of the *S. cerevisiae* TAM strain as a template [21, 39]. Subsequent DNA editing in this Pdc^−^ background proved difficult. To facilitate subsequent modifications, the *PDC1* gene was re-introduced in a manner which allowed its rapid excision. The *PDC1* gene (including native promoter and terminator) was amplified from CEN.PK113-7D using primers with added homology to the coding region of the *ADE2* locus and the *TEF2* terminator region of the *amdS* cassette. The *amdS* gene cassette was amplified in a similar manner from pUG-AmdSYM [40] using primers with added homology to the target region of the *ADE2* locus. Transformation of both fragments into CEN.PK113-5D resulted in simultaneous assembly and integration of both cassettes at the *ADE2* locus. Correct integrants were selected by plating transformants on medium containing 0.6 g/L acetamide as the sole nitrogen source [40], 20 mg/L adenine to relieve the auxotrophy caused by *ADE2* disruption (which allowed for detection of integrants by their red colony colour) [41], and 0.15 g/L uracil to complement the uracil auxotrophy. This resulted in strain IMI271. While integration of the *PDC1*-*amdS* cassette interrupted the *ADE2* gene, its full sequence information was retained in the genome. A functional *ADE2* gene and adenine prototrophy could therefore be restored by excision of the cassette ([42], Fig. 2b). The integrated cassette was then used as PCR template to generate a full *PDC1*-*amdS* integration cassette at the *ADE2* locus, the resulting cassette was then transformed into IMI244 (*pdc1*Δ, *pdc5*Δ, *pdc6*Δ *MTH1ΔT*), using the selection procedure described above, resulting in strain IMI275. To allow for subsequent rapid deletions in this strain background, the CRISPR-Cas gene editing system was introduced [43]. A *cas9*-natNT2 gene cassette was generated by amplifying the *cas9* overexpression cassette from p414-pTEF1-*cas9*-tCYC [44] using primers with added homology to the upstream region of the *PDC6* locus and a unique 60 bp tag (Cas9-PDC6 homology fwd/Cas9-tag homology rev). Similarly, the natNT2 expression cassette was amplified from pUG-natNT2 [45] using primers with added homology to the downstream region of the *PDC6* locus and the same unique 60 bp tag (natNT2-tag homology fwd/natNT2-PDC6 homology rev) (Table 7). After transformation of both fragments, selection on agar medium containing 100 mg/L nourseothricin (Jena Bioscience, Jena, Germany), yielded strain IMI302. Finally, the decarboxylase-negative strain IMK647 was constructed by removing the *ARO10* gene using the introduced Cas9 system. Assembly of a plasmid containing the *ARO10*-specific guide RNA and subsequent Cas9-mediated removal of the *ARO10* gene were achieved in a single in vivo homologous recombination reaction step [43]. In this step, transformation of the CRISPR plasmid backbone (amplified from pROS10), the *ARO10* specific guide RNA fragment and the single-stranded *ARO10* repair fragments resulted in in vivo assembly of the plasmid, a Cas9-mediated double-strand break in the *ARO10* gene, and repair of that break using a repair fragment with homology to the upstream and downstream regions of *ARO10*. Transformants were selected on SMG plates supplemented with 20 mg/L adenine. A transformant with the correct genotype was then restreaked three times on plates containing 2.3 g/L fluoroacetamine, 1 g/L 5-fluoorotic acid (5′FOA), 0.150 g/L uracil, and the absence of adenine to induce the simultaneous loss of the in vivo assembled plasmid containing the *ARO10*-specific guide RNA and the *PDC1*-*amdS* cassette (Fig. 2b) resulting in strain IMK647.

The 2-oxo-acid decarboxylase overexpression strains, as well as the prototrophic 2-oxo acid decarboxylase minus empty vector control were constructed by transforming IMK647 with plasmids p426GPD (empty vector), pUDE001 (*ARO10*), pUDE321 (*kdcA*) and pUDE336 (*kivD*), resulting in strains IME259 (p426GPD), IME260 (*ARO10*), IME261 (*kdcA*) and IME262 (*kivD*). An overview of the different steps in the construction of these strains and the use of the removable *PDC1*-*amdS* cassette is presented in Fig. 2. After propagation in *E. coli*, plasmids were isolated with the Sigma GenElute Plasmid kit (Sigma Aldrich). In all cases yeast transformants were selected on SME agar.

*Saccharomyces cerevisiae* transformation was performed by the lithium acetate method [46]. Correct assembly of plasmids and chromosomal integration constructs were checked by diagnostic PCR with specific primer sets (Table 7), using DreamTaq polymerase (Thermo scientific) and desalted primers (Sigma Aldrich).

### Shake flask cultivation

All *S. cerevisiae* strains were grown in chemically defined medium as described above. Strains were grown in either 1-L or 500-mL shake flasks containing 200 or 100 mL synthetic medium, respectively, at 30 °C in an Innova incubator (New Brunswick Scientific, Edison, NJ) set at 200 rpm. Optical density at 660 nm was measured using a Libra S11 spectrophotometer (Biochrom, Cambridge, UK).

### In vitro enzymatic analysis of 2-oxo acid decarboxylase overexpression

Determination of *K*_m_ and *V*_max_ of 2-oxo acid decarboxylases was determined in an enzyme assay in which the activity of a 2-oxo acid decarboxylase was coupled to the conversion of an aldehyde to its corresponding acid by purified *S. cerevisiae* aldehyde dehydrogenase [13]. NAD^+^ reduction was monitored spectrophotometrically at 340 nm. Assays were performed at 30 °C in a Hitachi U-3010 spectrophotometer. Cell extracts were prepared by harvesting 62.5 mg of biomass by centrifugation at 4600×*g* for 5 min. Cell pellets were washed with 10 mM potassium phosphate buffer containing 2 mM EDTA at pH 7.5, then washed again and resuspended in 100 mM potassium phosphate buffer at pH 7.5 containing 2 mM MgCl_2_ and 2 mM dithiothreitol (DTT). Extracts were prepared using Fast Prep FP120 (Thermo Scientific) with 0.7-mm glass beads. Cells were disintegrated in four bursts of 20 s with 30 s of cooling on ice between runs. Cellular debris was removed by centrifugation at 47,000*g* for 20 min at 4 °C [47]. The cell extract was then used immediately for enzyme assays. Protein concentrations in cell extracts were determined with the Lowry method [48].

The 1 mL assay mixture for measuring 2-oxo acid decarboxylase activity contained 100 mM potassium phosphate buffer (pH 7.0), 0.2 mM thiamine pyrophosphate, 5 mM MgCl_2_, 15 mM pyrazole, 2 mM NAD^+^ and 1.75 U/mL aldehyde dehydrogenase and between 5 and 100 µL of cell extract. The reaction was initiated by addition of a 2-oxo acid. Reaction rates were linearly proportional to the amount of cell extract added. Enzyme activities were assayed at substrate concentrations ranging from 0 to 12.5 mM for phenylpyruvate, 0–100 mM for pyruvate, and 0–50 mM for α-ketoisovalerate, α-ketomethyvalerate, α-ketoisocaproate, 4-methylthio-2-oxobutanoate, 2-oxobutanoate and 2-oxopentanoate. K_m_ and V_max_ were estimated by fitting kinetic data from at least six different substrate concentrations with GraphPad Prism 4.0 (GraphPad Software, Inc, La Jolla, CA) using non-linear regression of the Michaelis–Menten and Hill equations.

### 2-oxo acid decarboxylase-dependent restoration of amino acid catabolism

Restoration of amino acid catabolism by various 2-oxo acid decarboxylases was tested by measuring the specific growth rate in micro-titre plate (µ_MTP_) of strains incubated aerobically in 48-well plates (Greiner Bio-One, Alphen aan Den Rijn, The Netherlands) with different amino acids as sole nitrogen source. Cells were pre-cultured in 100 mL SME medium containing 0.2 mM (NH_4_)_2_SO_4_. Precultures were grown until the residual ammonium was depleted. Cells were then washed twice in nitrogen- and carbon-source-free synthetic medium, and inoculated in wells to an initial OD 660 of 0.01. Each well contained 500 µL SMG medium supplemented with 5 g/L of either valine, leucine, isoleucine, phenylalanine, methionine or (NH_4_)_2_SO_4_. The 48-well plates were incubated aerobically at 30 °C in a GENios pro plate reader (Tecan Benelux, Giessen, The Netherlands), under constant shaking at 200 rpm. OD 660 was measured automatically at 15 min intervals.

### In vivo α-ketoisovalerate bioconversion by 2-oxo acid decarboxylases

The in vivo activity and affinity of each 2-oxo acid decarboxylase towards α-ketoisovalerate (KIV) was assessed by measuring the production of isobutanol in micro-aerobic high-cell-density cultures supplemented with high concentrations of KIV. To this end, each strain was pregrown in 200 mL SME medium until mid-exponential phase (~OD 4.0), cells were centrifuged 4700*g* for 5 min and resuspended to a final OD 660 of ~40.0 in 25 mL synthetic medium supplemented with 10 g/L glucose Tween-80 (420 mg/L), ergosterol (10 mg/L) and 100 mM KIV. The initial pH was set to 3.5 or 6.0 by addition of 2 M H_2_SO_4_ or 2 M KOH. Cultures were then incubated at 30 °C in 30 mL rubber-stoppered serum bottles to create micro-aerobic conditions. Each rubber stopper was pierced with a 0.6 mm, Microlance needle (Becton–Dickinson, Breda, The Netherlands), fitted with a cotton-wool plugged syringe cylinder to prevent pressure build-up. Samples were taken to determine extracellular metabolite concentrations, OD 660 and pH over the linear phase of glucose consumption. To limit introduction of oxygen, samples were taken by attaching a sterile syringe to the needle, inverting the serum bottle and withdrawing ~200 µL culture liquid. To determine extracellular glucose, ethanol, KIV (α-ketoisovalerate) and isobutanol concentrations culture samples were spun down at 3500 g and the supernatant was collected. Extracellular metabolites were analysed using an Agilent 1260 Affinity HPLC machine (Agilent Technologies, Amstelveen, The Netherlands) with an Aminex HPX-87H ion-exchange column (BioRad) operated at 60 °C with a mobile phase of 5 mM H_2_SO_4_ and a flow rate of 0.6 mL/min. Biomass concentrations were estimated from OD 660 measurements, assuming that 1 g/L of cell biomass corresponds to an OD 660 value of 4.02.

## Additional file

10.1186/s13068-015-0374-0 Sequence of the expression cassettes of the yeast codon optimised kdcA and kivdD genes.

## Abbreviations

KIV: α-ketoisovalerate
Pdc: pyruvate decarboxylase
Pdc^−^: pyruvate decarboxylase negative
OD: optical density
SMG: chemically defined (synthetic) medium glucose
SME: chemically defined (synthetic) medium ethanol
BLAST: Basic Local Alignment Search Tool
5’FOA: 5-fluoorotic acid
DTT: dithiothreitol
Val: valine
Ile: isoleucine
Leu: leucine
Met: methionine
