# Supplementary material for: Comparative assessment of native and heterologous 2-oxo acid decarboxylases for application in isobutanol production by Saccharomyces cerevisiae
Source: Biotechnol Biofuels. 2015 Dec 1;8:204. doi: 10.1186/s13068-015-0374-0 (PMC4665922; doi:10.1186/s13068-015-0374-0)
Supplement: Supplementary file 1 — 10.1186/s13068-015-0374-0 Sequence of the expression cassettes of the yeast codon optimised kdcA and kivdD genes. [file 13068_2015_374_MOESM1_ESM.docx]

**Supplemental material**

Sequence of the expression cassettes of the yeast codon optimised kdcA and kivdD genes.

*TDH3p-kdcA-CYC1t*

TCATTATCAATACTCGCCATTTCAAAGAATACGTAAATAATTAATAGTAGTGATTTTCCTAACTTTATTTAGTCAAAAAATTAGCCTTTTAATTCTGCTGTAACCCGTACATGCCCAAAATAGGGGGCGGGTTACACAGAATATATAACATCGTAGGTGTCTGGGTGAACAGTTTATTCCTGGCATCCACTAAATATAATGGAGCCCGCTTTTTAAGCTGGCATCCAGAAAAAAAAAGAATCCCAGCACCAAAATATTGTTTTCTTCACCAACCATCAGTTCATAGGTCCATTCTCTTAGCGCAACTACAGAGAACAGGGGCACAAACAGGCAAAAAACGGGCACAACCTCAATGGAGTGATGCAACCTGCCTGGAGTAAATGATGACACAAGGCAATTGACCCACGCATGTATCTATCTCATTTTCTTACACCTTCTATTACCTTCTGCTCTCTCTGATTTGGAAAAAGCTGAAAAAAAAGGTTGAAACCAGTTCCCTGAAATTATTCCCCTACTTGACTAATAAGTATATAAAGACGGTAGGTATTGATTGTAATTCTGTAAATCTATTTCTTAAACTTCTTAAATTCTACTTTTATAGTTAGTCTTTTTTTTAGTTTTAAAACACCAGAACTTAGTTTCGACGGATATGTACACTGTTGGTGACTACTTGTTGGACAGATTGCACGAATTGGGTATCGAAGAAATCTTCGGTGTTCCAGGTGACTACAACTTGCAATTCTTGGACCAAATCATCTCTAGAGAAGACATGAAGTGGATCGGTAACGCTAACGAATTGAACGCTTCTTACATGGCTGACGGTTACGCTAGAACTAAGAAGGCTGCTGCTTTCTTGACTACTTTCGGTGTTGGTGAATTGTCTGCTATCAACGGTTTGGCTGGTTCTTACGCTGAAAACTTGCCAGTTGTTGAAATCGTTGGTTCTCCAACTTCTAAGGTTCAAAACGACGGTAAGTTCGTTCACCACACTTTGGCTGACGGTGACTTCAAGCACTTCATGAAGATGCACGAACCAGTTACTGCTGCTAGAACTTTGTTGACTGCTGAAAACGCTACTTACGAAATCGACAGAGTTTTGTCTCAATTGTTGAAGGAAAGAAAGCCAGTTTACATCAACTTGCCAGTTGACGTTGCTGCTGCTAAGGCTGAAAAGCCAGCTTTGTCTTTGGAAAAGGAATCTTCTACTACTAACACTACTGAACAAGTTATCTTGTCTAAGATCGAAGAATCTTTGAAGAACGCTCAAAAGCCAGTTGTTATCGCTGGTCACGAAGTTATCTCTTTCGGTTTGGAAAAGACTGTTACTCAATTCGTTTCTGAAACTAAGTTGCCAATCACTACTTTGAACTTCGGTAAGTCTGCTGTTGACGAATCTTTGCCATCTTTCTTGGGTATCTACAACGGTAAGTTGTCTGAAATCTCTTTGAAGAACTTCGTTGAATCTGCTGACTTCATCTTGATGTTGGGTGTTAAGTTGACTGACTCTTCTACTGGTGCTTTCACTCACCACTTGGACGAAAACAAGATGATCTCTTTGAACATCGACGAAGGTATCATCTTCAACAAGGTTGTTGAAGACTTCGACTTCAGAGCTGTTGTTTCTTCTTTGTCTGAATTGAAGGGTATCGAATACGAAGGTCAATACATCGACAAGCAATACGAAGAATTCATCCCATCTTCTGCTCCATTGTCTCAAGACAGATTGTGGCAAGCTGTTGAATCTTTGACTCAATCTAACGAAACTATCGTTGCTGAACAAGGTACTTCTTTCTTCGGTGCTTCTACTATCTTCTTGAAGTCTAACTCTAGATTCATCGGTCAACCATTGTGGGGTTCTATCGGTTACACTTTCCCAGCTGCTTTGGGTTCTCAAATCGCTGACAAGGAATCTAGACACTTGTTGTTCATCGGTGACGGTTCTTTGCAATTGACTGTTCAAGAATTGGGTTTGTCTATCAGAGAAAAGTTGAACCCAATCTGTTTCATCATCAACAACGACGGTTACACTGTTGAAAGAGAAATCCACGGTCCAACTCAATCTTACAACGACATCCCAATGTGGAACTACTCTAAGTTGCCAGAAACTTTCGGTGCTACTGAAGACAGAGTTGTTTCTAAGATCGTTAGAACTGAAAACGAATTCGTTTCTGTTATGAAGGAAGCTCAAGCTGACGTTAACAGAATGTACTGGATCGAATTGGTTTTGGAAAAGGAAGACGCTCCAAAGTTGTTGAAGAAGATGGGTAAGTTGTTCGCTGAACAAAACAAGTAAGATTAATATAATTATATAAAAATATTATCTTCTTTTCTTTATATCTAGTGTTATGTAAAATAAATTGATGACTACGGAAAGCTTTTTTATATTGTTTCTTTTTCATTCTGAGCCACTTAAATTTCGTGAATGTTCTTATAAGGGACGGTAGATTTACAAGTGATACAACAAAAAGCAAGGCGCTTTTTCTAATAAAAAGAAGAAAAGCATTTAACAATTGAACACCTCTATATCAACGAAGAATATTACTTTGTCTCTAAATCCTTGTAAAATGTGTACGATCTCTATATGGGTTACTCAGAAGTGTACCGAAGACTGCATTGAAAGTTTATGTTTTTTCACTGCAAGCGTCATTTTCGC

*TDH3p-kivD-CYC1t*

AGTTTATCATTATCAATACTCGCCATTTCAAAGAATACGTAAATAATTAATAGTAGTGATTTTCCTAACTTTATTTAGTCAAAAAATTAGCCTTTTAATTCTGCTGTAACCCGTACATGCCCAAAATAGGGGGCGGGTTACACAGAATATATAACATCGTAGGTGTCTGGGTGAACAGTTTATTCCTGGCATCCACTAAATATAATGGAGCCCGCTTTTTAAGCTGGCATCCAGAAAAAAAAAGAATCCCAGCACCAAAATATTGTTTTCTTCACCAACCATCAGTTCATAGGTCCATTCTCTTAGCGCAACTACAGAGAACAGGGGCACAAACAGGCAAAAAACGGGCACAACCTCAATGGAGTGATGCAACCTGCCTGGAGTAAATGATGACACAAGGCAATTGACCCACGCATGTATCTATCTCATTTTCTTACACCTTCTATTACCTTCTGCTCTCTCTGATTTGGAAAAAGCTGAAAAAAAAGGTTGAAACCAGTTCCCTGAAATTATTCCCCTACTTGACTAATAAGTATATAAAGACGGTAGGTATTGATTGTAATTCTGTAAATCTATTTCTTAAACTTCTTAAATTCTACTTTTATAGTTAGTCTTTTTTTTAGTTTTAAAACACCAGAACTTAGTTTCGACGGATATGTACACTGTTGGTGACTACTTGTTGGACAGATTGCACGAATTGGGTATCGAAGAAATCTTCGGTGTTCCAGGTGACTACAACTTGCAATTCTTGGACCAAATCATCTCTCACAAGGACATGAAGTGGGTTGGTAACGCTAACGAATTGAACGCTTCTTACATGGCTGACGGTTACGCTAGAACTAAGAAGGCTGCTGCTTTCTTGACTACTTTCGGTGTTGGTGAATTGTCTGCTGTTAACGGTTTGGCTGGTTCTTACGCTGAAAACTTGCCAGTTGTTGAAATCGTTGGTTCTCCAACTTCTAAGGTTCAAAACGAAGGTAAGTTCGTTCACCACACTTTGGCTGACGGTGACTTCAAGCACTTCATGAAGATGCACGAACCAGTTACTGCTGCTAGAACTTTGTTGACTGCTGAAAACGCTACTGTTGAAATCGACAGAGTTTTGTCTGCTTTGTTGAAGGAAAGAAAGCCAGTTTACATCAACTTGCCAGTTGACGTTGCTGCTGCTAAGGCTGAAAAGCCATCTTTGCCATTGAAGAAGGAAAACTCTACTTCTAACACTTCTGACCAAGAAATCTTGAACAAGATCCAAGAATCTTTGAAGAACGCTAAGAAGCCAATCGTTATCACTGGTCACGAAATCATCTCTTTCGGTTTGGAAAAGACTGTTACTCAATTCATCTCTAAGACTAAGTTGCCAATCACTACTTTGAACTTCGGTAAGTCTTCTGTTGACGAAGCTTTGCCATCTTTCTTGGGTATCTACAACGGTACTTTGTCTGAACCAAACTTGAAGGAATTCGTTGAATCTGCTGACTTCATCTTGATGTTGGGTGTTAAGTTGACTGACTCTTCTACTGGTGCTTTCACTCACCACTTGAACGAAAACAAGATGATCTCTTTGAACATCGACGAAGGTAAGATCTTCAACGAAAGAATCCAAAACTTCGACTTCGAATCTTTGATCTCTTCTTTGTTGGACTTGTCTGAAATCGAATACAAGGGTAAGTACATCGACAAGAAGCAAGAAGACTTCGTTCCATCTAACGCTTTGTTGTCTCAAGACAGATTGTGGCAAGCTGTTGAAAACTTGACTCAATCTAACGAAACTATCGTTGCTGAACAAGGTACTTCTTTCTTCGGTGCTTCTTCTATCTTCTTGAAGTCTAAGTCTCACTTCATCGGTCAACCATTGTGGGGTTCTATCGGTTACACTTTCCCAGCTGCTTTGGGTTCTCAAATCGCTGACAAGGAATCTAGACACTTGTTGTTCATCGGTGACGGTTCTTTGCAATTGACTGTTCAAGAATTGGGTTTGGCTATCAGAGAAAAGATCAACCCAATCTGTTTCATCATCAACAACGACGGTTACACTGTTGAAAGAGAAATCCACGGTCCAAACCAATCTTACAACGACATCCCAATGTGGAACTACTCTAAGTTGCCAGAATCTTTCGGTGCTACTGAAGACAGAGTTGTTTCTAAGATCGTTAGAACTGAAAACGAATTCGTTTCTGTTATGAAGGAAGCTCAAGCTGACCCAAACAGAATGTACTGGATCGAATTGATCTTGGCTAAGGAAGGTGCTCCAAAGGTTTTGAAGAAGATGGGTAAGTTGTTCGCTGAACAAAACAAGTCTTAAGATTAATATAATTATATAAAAATATTATCTTCTTTTCTTTATATCTAGTGTTATGTAAAATAAATTGATGACTACGGAAAGCTTTTTTATATTGTTTCTTTTTCATTCTGAGCCACTTAAATTTCGTGAATGTTCTTATAAGGGACGGTAGATTTACAAGTGATACAACAAAAAGCAAGGCGCTTTTTCTAATAAAAAGAAGAAAAGCATTTAACAATTGAACACCTCTATATCAACGAAGAATATTACTTTGTCTCTAAATCCTTGTAAAATGTGTACGATCTCTATATGGGTTACTCAGAAGTGTACCGAAGACTGCATTGAAAGTTTATGTTTTTTCACTGCAAGCGTCATTTTCGC
